# Exploring the effects of depression and treatment of depression in reinforcement learning

**DOI:** 10.3389/fnint.2013.00072

**Published:** 2013-10-09

**Authors:** Pedro Castro-Rodrigues, Albino J. Oliveira-Maia

**Affiliations:** ^1^Champalimaud Neuroscience Programme, Champalimaud FoundationLisboa, Portugal; ^2^Neuropsychiatry Unit, Champalimaud Clinical Centre, Champalimaud FoundationLisboa, Portugal; ^3^Centro Hospitalar Psiquiátrico de LisboaLisboa, Portugal; ^4^Department of Psychiatry and Mental Health, Centro Hospitalar de Lisboa OcidentalLisboa, Portugal

**Keywords:** depression, SSRI antidepressants, reinforcement (Psychology), punishment, reward

In this research topic, Herzallah et al. (2013) have contributed original work, exploring the effects of Major Depressive Disorder (MDD) and of treatment with a selective serotonin reuptake inhibitor (SSRI) on responses in a reinforcement-learning task. The task developed by the authors allows for dissociation between positive (reward) and negative (punishment) reinforcement learning and was used to compare, in a cross-sectional design, healthy controls (HC), patients with MDD prior to initiating treatment (MDD), and patients recovered from MDD under treatment with paroxetine (a SSRI; MDD-T). The main finding of their work was that, when compared to HC, patients with untreated MDD were impaired in reward learning, while MDD patients under treatment with paroxetine were impaired in both reward and punishment learning. As a result of these response profiles, the relative learning from reward and punishment was similar between HC and MDD-T (due to blunted responses to both valences in MDD-T) while, in MDD patients, learning from negative feedback was exaggerated when compared to learning from positive reinforcement.

The findings reported in this paper (Herzallah et al., 2013) contribute novel insights to the body of literature exploring the relationship between depression, reinforcement and serotonin (5-HT). The interest on understanding reinforcement processes in the context of depression is sustained on the fact that anhedonia, i.e., the loss of interest or pleasure in all or almost all activities, is a key-symptom of MDD (APA Task Force on DSM-IV, 1994). Preclinical analogs to anhedonia, commonly used to assess depression-like behavior in rodents, are the sucrose intake and preference tests, whereby decreased intake of, or preference for, a sweet sucrose solution (relative to water) are argued to reflect an anhedonic state (Monleon et al., 1995). The relationship between serotonergic processing and depression has been suggested mostly from the anti-depressant effects of drugs modulating serotonergic neurotransmission (Shopsin et al., 1976; Delgado et al., 1990). Recently, several hypotheses have been put forward to suggest that the link between MDD and 5HT might be indirect and mediated by associative learning (Robinson and Sahakian, 2008) and/or disinhibition of negative thoughts (Dayan and Huys, 2008). Elliott et al. (1996), for example, found that during application of a neuropsychological assessment battery, after having solved one problem incorrectly, MDD patients were more likely than controls to fail the subsequent problem. This finding correlated with the severity of the depression and has been interpreted as oversensitivity to negative feedback.

Several paradigms have been used to explore the processing capabilities of depressed patients in contexts of reward and/or punishment. Findings have not been entirely consistent, possibly due to heterogeneity of patient samples and differences in testing protocols, with different authors suggesting that patients with MDD have more significant deficits in reward learning (Henriques and Davidson, 2000; Pizzagalli et al., 2008; McFarland and Klein, 2009; Robinson et al., 2012) punishment learning (Murphy et al., 2003; Santesso et al., 2008) or both (Must et al., 2006). Henriques and Davidson (2000) compared MDD patients to a group of non-depressed control subjects on a verbal memory task under three monetary payoff conditions: neutral, reward, and punishment. While control subjects maximized their earnings by modulating their pattern of response in both reward and punishment conditions, relative to the neutral condition, depressed subjects did not do so during reward. Others (McFarland and Klein, 2009) have reported similar findings, with reactivity in response to anticipated reward being significantly diminished in currently depressed compared with never depressed participants, and marginally diminished in comparison with previously depressed participants. There is also evidence to suggest exaggerated responses to punishment in MDD, rather than diminished responses to reward. In a study based on a probability reversal task (Murphy et al., 2003), when given misleading negative feedback, MDD patients were impaired in the ability to maintain the response set, as shown by their increased tendency to switch responding to the 'incorrect' stimulus following negative reinforcement, relative to controls.

Others have compared neural responses to stimuli with positive and negative valence between depressed and non-depressed volunteers. McCabe et al. (2009) compared unmedicated patients with a history of major depression to age and gender matched HC. Despite no differences in stimulus ratings, recovered patients had decreased neural responses to the pleasant stimulus in the ventral striatum and increased responses in the caudate nucleus to the aversive stimulus. The same authors (McCabe et al., 2010) later found that citalopram (a SSRI antidepressant), but not reboxetine (another antidepresssant that acts as a noradrenaline reuptake inhibitor), reduced activation from rewarding stimuli in the ventral striatum and orbitofrontal cortex. Citalopram also decreased neural responses to the aversive stimuli conditions in areas such as the lateral orbitofrontal cortex, while reboxetine produced a similar, although weaker, effect. Others have found that another antidepressant (duloxetine, a dual serotonin and noradrenaline reuptake inhibitor), rather than diminishing the neural processing of both rewarding and aversive stimuli, can increase ventral striatal activity in humans (Ossewaarde et al., 2011).

The findings now reported by Herzallah et al. (2013) highlight the importance of considering treatment effects when testing the relationship between depression and reinforcement learning. Furthermore, the authors provide behavioral support for the neural findings previously described by McCabe et al. (McCabe et al., 2010): in both cases responses to reward and punishment were reduced by treatment with a SSRI, possibly accounting for the experience of *emotional blunting described* by some patients during SSRI treatment (Price et al., 2009). However, as is acknowledged by the authors (Herzallah et al., 2013), these findings should be interpreted in the context of their cross-sectional study design. For example, it is unclear if, before starting treatment, disease severity in MDD-T patients was similar to that found in MDD patients, and also if the latter group will respond to treatment similarly to the MDD-T group. Importantly, because MDD-T patients are responders to treatment, the experimental design also does not distinguish entirely between the effects of paroxetine *per se* and the effects of recovery from depression. Future work, with a longitudinal study design and/or including groups at different phases of treatment or treated with non-SSRI drugs or non-pharmacological alternatives, should address these questions.

## References

[B1] American Psychiatric Association Task Force on DSM-IV. (1994). Diagnostic and Statistical Manual of Mental Disorders: DSM-IV. Washington, DC: American Psychiatric Association

[B2] Dayan P. Huys Q. J. (2008). Serotonin, inhibition and negative mood. PLoS Comput. Biol. 4:e4 10.1371/journal.pcbi.0040004 18248087 PMC2222921

[B3] Delgado P. L. Charney D. S. Price L. H. Aghajanian G. L. Landis H. Heninger G. R. (1990). Serotonin function and the mechanism of antidepressant action: reversal of antidepressant-induced remission by rapid depletion of plasma tryptophan. Arch. Gen. Psychiatry 47, 411–418 10.1001/archpsyc.1990.01810170011002 2184795

[B4] Elliott R. Sahakian B. J. McKay A. P. Herrod J. J. Robbins T. W. Paykel E. S. (1996). Neuropsychological impairments in unipolar depression: the influence of perceived failure on subsequent performance. Psychol. Med. 26, 975–989 10.1017/S0033291700035303 8878330

[B5] Henriques J. B. Davidson R. J. (2000). Decreased responsiveness to reward in depression. Cogn. Emot. 14, 711–724 10.1080/02699930050117684

[B6] Herzallah M. M. Moustafa A. A. Natsheh J. Y. Abdellatif S. M. Taha M. B. Tayem Y. I. (2013). Learning from negative feedback in patients with major depressive disorder is attenuated by SSRI antidepressants. Front. Integr. Neurosci. 7:67 10.3389/fnint.2013.00067 24065894 PMC3779792

[B7] McCabe C. Cowen P. J. Harmer C. J. (2009). Neural representation of reward in recovered depressed patients. Psychopharmacology 205, 667–677 10.1007/s00213-009-1573-9 19529923 PMC2718193

[B8] McCabe C. Mishor Z. Cowen P. J. Harmer C. J. (2010). Diminished neural processing of aversive and reward stimuli during selective serotonin reuptake inhibitor treatment. Biol. Psychiatry 67, 439–445 10.1016/j.biopsych.2009.11.001 20034615 PMC2828549

[B9] McFarland B. R. Klein D. N. (2009). Emotional reactivity in depression: diminished responsiveness to anticipated reward but not to anticipated punishment or to nonreward or avoidance. Depress. Anxiety 26, 117–122 10.1002/da.20513 18972567

[B10] Monleon S. D'Aquila P. Parra A. Simon V. M. Brain P. F. Willner P. (1995). Attenuation of sucrose consumption in mice by chronic mil stress and its restoration by imipramine. Psychopharmacology (Berl.) 117, 453–547 7604147 10.1007/BF02246218

[B11] Murphy F. C. Michael A. Robbins T. W. Sahakian B. J. (2003). Neuropsychological impairment in patients with major depressive disorder: the effects of feedback on task performance. Psychol. Med. 33, 455–467 10.1017/S0033291702007018 12701666

[B12] Must A. Szabó Z. Bódi N. Szász A. Janka Z. Kéria S. (2006). Sensitivity to reward and punishment and the prefrontal cortex in major depression. J. Affect. Disord. 90, 209–215 10.1016/j.jad.2005.12.005 16412520

[B13] Ossewaarde L. O. Verkles R. J. Hermans E. J. Kooijmana S. C. Urnera M. Tendolkar (2011). Two-week administration of the combined serotonin-noradrenaline reuptake inhibitor duloxetine augments functioning of mesolimbic incentive processing circuits. Biol. Psychiatry 70, 568–574 10.1016/j.biopsych.2011.03.041 21601833

[B14] Pizzagalli D. A. Iosifescu D. Hallett L. A. Ratner K. G. Fava M. (2008). Reduced hedonic capacity in major depressive disorder: evidence from a probabilistic reward task. J. Psychiatr. Res. 43, 76–87 10.1016/j.jpsychires.2008.03.001 18433774 PMC2637997

[B15] Price J. Cole V. Goodwin G. M. (2009). Emotional side-effects of selective serotonin reuptake inhibitors: qualitative study. Br. J. Psychiatry 195, 211–217 10.1192/bjp.bp.108.051110 19721109

[B17] Robinson O. J. Cools R. Carlisi C. O. Sahakian B. J. Drevets W. C. (2012). Ventral striatum response during reward and punishment reversal learning in unmedicated major depressive disorder. Am. J. Psychiatry 169, 152–159 10.1176/appi.ajp.2011.11010137 22420038 PMC5648982

[B18] Robinson O. J. Sahakian B. J. (2008). Recourrence in major depressive disorder: a neurocognitive perspective. Psychol. Med. 38, 315–318 10.1017/S0033291707001249 18298875

[B19] Santesso D. L. Steele K. T. Bogdan R. Holmes A. J. Deveney C. M. Meites T. M. (2008). Enhanced negative feedback responses in remitted depression. Neuroreport 19, 1045–1048 10.1097/WNR.0b013e3283036e73 18580576 PMC3034237

[B20] Shopsin B. Friedman E. Gershon S. (1976). Parachlorophenylalanine reversal of tranylcypromine effects in depressed patients. Arch. Gen. Psychiatry 33, 811–819 10.1001/archpsyc.1976.01770070041003 133650

